# Pregnancy, prison and perinatal outcomes in New South Wales, Australia: a retrospective cohort study using linked health data

**DOI:** 10.1186/1471-2393-14-214

**Published:** 2014-06-27

**Authors:** Jane R Walker, Lisa Hilder, Michael H Levy, Elizabeth A Sullivan

**Affiliations:** 1 National Perinatal Epidemiology and Statistics Unit, School of Women’s and Children’s Health, Faculty of Medicine, University of New South Wales, Randwick Hospitals Campus, Level 2, McNevin Dickson Building, Randwick 2031, NSW, Australia; 2 College of Medicine, Biology and Environment, Australian National University, Canberra, Australia; 3 Faculty of Health, University of Technology Sydney, Broadway Campus, NSW, Australia

**Keywords:** Maternal morbidity, Perinatal outcome, Women in prison, Incarceration in pregnancy, Prison health care, Antenatal care, Substance use, Mental health disorder

## Abstract

**Background:**

Studies from the United States and the United Kingdom have found that imprisoned women are less likely to experience poorer maternal and perinatal outcomes than other disadvantaged women. This population-based study used both community controls and women with a history of incarceration as a control group, to investigate whether imprisoned pregnant women in New South Wales, Australia, have improved maternal and perinatal outcomes.

**Methods:**

Retrospective cohort study using probabilistic record linkage of routinely collected data from health and corrective services in New South Wales, Australia. Comparison of the maternal and perinatal outcomes of imprisoned pregnant women aged 18–44 years who gave birth between 2000–2006 with women who were (i) imprisoned at a time other than pregnancy, and (ii) community controls. Outcomes of interest: onset of labour, method of birth, pre-term birth, low birthweight, Apgar score, resuscitation, neonatal hospital admission, perinatal death.

**Results:**

Babies born to women who were imprisoned during pregnancy were significantly more likely to be born pre-term, have low birthweight, and be admitted to hospital, compared with community controls. Pregnant prisoners did not have significantly better outcomes than other similarly disadvantaged women (those with a history of imprisonment who were not imprisoned during pregnancy).

**Conclusions:**

In contrast to the published literature, we found no evidence that contact with prison health services during pregnancy was a “therapunitive” intervention. We found no association between imprisonment during pregnancy and improved perinatal outcomes for imprisoned women or their neonates. A history of imprisonment remained the strongest predictor of poor perinatal outcomes, reflecting the relative health disadvantage experienced by this population of women.

## Background

New South Wales (NSW) has the largest overall population of the Australian states and territories. The state accounts for almost one-third of Australia’s births annually and 30% of the Australian female prisoner population [1]. In NSW and Australia as a whole, the majority of imprisoned women are of childbearing age. Women in NSW prisons typically experience a series of short sentences and/or periods of remand, which constitutes ‘a form of serial institutionalisation’ [2]. The average daily number of women in full-time custody in NSW in 2012 was 661 [3]. Most are held in three female-only institutions, with smaller numbers in female-only units, work camps and transitional centres. In the state of NSW, the standard term of reference for Indigenous Australians is Aboriginal peoples, ‘in recognition that Aboriginal people are the original inhabitants of NSW’ [4]. Aboriginal women are severely over-represented in the NSW prisoner population, comprising 30% of female prisoners in NSW [1] but only 2% of the general population. Their rate of imprisonment in NSW rose by 12.5% between 2006 and 2009 alone [5].

Women in prison have a higher number of pregnancies and a disproportionate number have given birth before the age of twenty, when compared with the general birthing population of NSW. The NSW Inmate Health Survey is the only source of data on the birthing histories of female prisoners in the State. Eighty-two percent of women who participated in the 2009 NSW Inmate Health Survey had been pregnant at least once, including 27% who reported having had five or more pregnancies [6]. In contrast, between 2002 and 2006, 1.6% of women in the general NSW population who gave birth had five or more previous pregnancies of at least 20 weeks duration [7]. Whilst the figures are not directly comparable, these published data also show that more than half (51%) of women in the NSW Inmate Health Survey with a history of childbirth gave birth before twenty years of age, whilst the proportion of pregnancies of at least 20 weeks duration in the NSW population which were to women aged less than twenty years was only 4% between 2002–2006 [6,7].

Studies in the United States (US) and United Kingdom (UK) have compared the maternity outcomes of women in prison with those of population control groups [8-10], and disadvantaged control groups [11,12]. Findings are equivocal, but a systematic review concluded that imprisoned women were more likely to experience poorer maternity outcomes than women in the general population, yet less likely to experience poorer outcomes than other disadvantaged women [13]. Studies have suggested that imprisonment provides safety from abusive relationships, better nutrition, improved access to health care, and cessation of drug and alcohol use [8-12]. If this is the case, improvements in outcomes for imprisoned women in NSW may be less evident, since they are imprisoned for shorter periods than their US counterparts. The mean length of stay in full-time custody for sentenced female prisoners in NSW is 196 days (or 300 days for remand to sentenced custody) [14], compared with 18 months (547 days) in US state prisons [15].

The aim of this study is to determine whether women imprisoned during pregnancy have improved perinatal outcomes, compared with women in the general community, and women who share similar socio-demographic characteristics including a history of incarceration (at a time other than during their pregnancy).

### Human research ethics committee approval

Human Research Ethics Committee approval for the study was provided by the NSW Population and Health Service Research Ethics Committee, Justice Health Research Ethics Committee, Corrective Services NSW Research Ethics Committee, the Aboriginal Health and Medical Research Council of NSW, and the University of New South Wales.

## Methods

### Study design

This retrospective cohort study used probabilistic record linkage of routinely collected data from health and corrective services in NSW of women aged 18–44 years who gave birth in NSW between 2000 and 2006. These were the most contemporaneous data available to the study. Maternal data from the NSW Midwives Data Collection (MDC) were linked with data from the Offender Integrated Management System (OIMS), NSW Admitted Patient Data Collection (APDC), Pharmaceutical Drugs of Addictions System (PHDAS); and with their baby data from the APDC. Further information about these routine data collections and the record linkage methodology is provided in Additional file 1. Following the provision of data for the study, in 2011 NSW Health Records and Information Privacy Act (2002) regulations were amended to effectively restrict the linkage of records belonging to the NSW Department of Health with those belonging to non-Health agencies.

### Study population

We selected the first singleton (index) birth during the study period for each woman, and grouped the women according to their prisoner status. We created three study groups:

#### ‘Prison pregnancies’

This group comprised women who were incarcerated for at least five consecutive days during their index pregnancy, and contributed 302 births. For the analysis of labour and delivery outcomes, we separated prison pregnancies into women who were imprisoned at the time of their birth episode (‘birthing prisoners’, n = 99) and women who were no longer incarcerated at the time of their birth episode (‘former pregnant prisoners’, n = 203). The prisoner status of these two sub-groups differed at the time of their birth admission, when these data were collected. This enabled us to examine whether there were differences in the rate of interventions in labour and delivery for prisoners and non-prisoners.

In this study, antenatal care for pregnant prisoners was provided under shared care arrangements with local hospital maternity units, where women were also admitted for the birth, if this coincided with their incarceration [16]. Additionally, the women were usually incarcerated at two major centres with comprehensive clinic facilities, which are provided by the NSW Department of Health; in close proximity to specialised community services. In a small number of cases pregnant women may be incarcerated for shorter periods at smaller regional centres with more limited access to services.

#### ‘Prison controls’

This group comprised women who were incarcerated for at least five days during the study period, but not during their index pregnancy, and contributed 1,238 births. This group functions as a ‘disadvantaged’ control group. These women share similar socio-demographic characteristics with the study group. This group was not exposed to imprisonment during pregnancy.

#### ‘Community controls’

This group of 39,367 births comprised a representative 10% sample of women giving birth for the first time in the study period, selected randomly from women in NSW who had no record of imprisonment of any duration between 1998 and 2006.

### Definitions

#### Imprisonment

Imprisonment in the context of this study was defined as a minimum of five consecutive days’ full-time detention at a NSW correctional facility. Police detention, periodic detention, and community sentences were excluded. A five-day threshold was selected, following consultation with prison health and corrective services service providers, for two reasons:

• Around 30% of women in NSW prisons are unsentenced, with an average length of stay in prison of around 30 days [1,17]. The study was designed to capture this large proportion of women serving short periods of remand custody.

• Justice Health has protocols for the detection and management of pregnancy in prisoners in NSW, which require that all women undergo pregnancy testing on reception to prison and at 28 days. Pregnant women undergo immediate assessments for mental health, self-harm risk, women’s health, and blood-borne viruses. Those with drug and alcohol issues are referred to the Drug and Alcohol Medical Officer and ongoing management plans are initiated. These immediate interventions alone could potentially offer significant health gains for women and neonates, even in the context of a brief incarceration.

#### Opioid Substitution Therapy (OST)

We flagged women as having a history of OST if they had one or more records from the Pharmaceutical Drugs of Addiction database, indicating that they had been authorised to receive OST.

#### Mental health admission

We flagged women as having a history of mental health admission if their records linked with:

• One or more hospital separations with specified ICD10-AM [18] codes indicating a mental health disorder or cognitive disability (F00-F09, F20-F99); or self-harm (X60-X84, Y10-Y19, Y87.0, Z91.5); or substance use (F11-F19, T40, T42, T43); or alcohol abuse (E24.4, F10, G31.2, G62.1, G72.1, I426, K29.2, K70, K86.0, O35.4, R78.0, T51, X45, X65, Y15, Y57.3, Y90, Y91, Z50.2, Z71.4, Z72.1); or

• One or more hospital separations specifying admission to a psychiatric facility.

#### Smoking

Information about smoking is collected for the MDC from self-report by women during pregnancy or at the time of childbirth.

#### Neonatal hospital admission

We flagged babies as having a neonatal admission if their records linked with one or more hospital separations with a neonatal condition (ICD10-AM [18] codes P00.00-P99.9), before the age of 28 days.

#### Parity

Parity was defined as the number of a woman’s previous pregnancies that resulted in a live birth or a fetal death of ≥20 weeks’ gestation or ≥400 g birthweight.

#### ‘Disadvantage’

It was not possible to measure ‘disadvantage’ directly in this study. However, several covariates in our data set have a well-documented association with socioeconomic disadvantage; namely, younger maternal age, Aboriginal and Torres Strait Islander origin, smoking during pregnancy, use of OST, mental health admission, and imprisonment [6,19]. Imprisonment itself is a strong indicator of socioeconomic disadvantage: two-thirds (67%) of women in the 2009 NSW Inmate Health Survey were unemployed in the 6 months before their imprisonment, and 44% had been unemployed for five years or longer [6].

### Outcome measures

The outcomes of interest were: antenatal care, onset of labour, method of birth, pre-term birth (<37 weeks), low birthweight (<2500 g), low 5-minute Apgar score (<7), higher order resuscitation (intubation and Intermittent Positive Pressure Respiration (IPPR); external cardiac massage and ventilation), neonatal admission to hospital, Special Care Nursery or Neonatal Intensive Care, and perinatal death.

### Statistical analysis

All statistical analyses were conducted using IBM SPSS 20.0 software [20].

#### Descriptive statistics

We used one-way analysis of variance (ANOVA) and Bonferroni tests to look for differences between the study and control groups for maternal age and parity. We used Pearson’s Chi-Square tests to compare groups for Aboriginal and Torres Strait Islander origin, country of birth (Australia or overseas), any cigarette smoking during pregnancy, OST and mental health or drug and alcohol disorders (Table 1). We used logistic regression to calculate the log odds of less antenatal care and maternal morbidity, controlling for the known confounding effects of maternal age, parity, smoking, Aboriginal and Torres Strait Islander origin and mental health and/or drug and alcohol disorder (Table 2). We used Pearson’s Chi-Square tests to compare study and control groups for labour and delivery outcomes (Table 3).

**Table 1:** Sociodemographic characteristics of women in the study (i)

		**Pregnancy in prison (ii)**											
		**Birth in prison**		**Birth out of prison**		**All pregnancy in prison (ii)**		**Prison controls (iii)**			**Community controls (iv)**		
		***n***	**%**	***n***	***%***	***n***	***%***	***n***	**%**	**P value (v)**	***n***	**%**	**P value (v)**
**Maternal age (years)**													
Mean						26.5		27.2			30.0		<0.001
Less than 20		6	6.1	11	5.4	17	5.6	108	8.7	0.0032	1540	3.9	<0.0001
20–24		34	34.3	93	45.8	127	42.1	382	30.9		6045	15.4	
25–29		32	32.3	52	25.6	84	27.8	373	30.1		11472	29.1	
30–34		20	20.2	34	16.7	54	17.9	252	20.4		12798	32.5	
35 and over		7	7.1	13	6.4	20	6.6	119	9.6		7414	18.8	
**Parity**													
None		30	30.3	57	28.1	87	28.8	410	33.1	0.5511	22061	56.0	<0.0001
One		29	29.3	52	25.6	81	26.8	337	27.2		9764	24.8	
Two		17	17.2	38	18.7	55	18.2	207	16.7		4792	12.2	
Three		10	10.1	26	12.8	36	11.9	138	11.1		1705	4.3	
Four or more		13	13.1	30	14.8	43	14.2	146	11.8		1006	2.6	
Missing/not stated		0	0.0	0	0.0	0	0.0	0	0.0		39		
**Parous**		69	69.7	146	71.9	215	71.2	828	66.9	0.1509	17267	43.9	<0.0001
**Aboriginal/Torres Strait Islander**		28	28.3	56	27.6	84	27.8	255	20.6	0.0068	863	2.2	<0.0001
**Born in Australia**		85	85.9	181	89.2	266	88.1	1091	88.1	0.9821	27598	70.1	<0.0001
**Opioid Substitution Therapy (OST)**		4	4.0	3	1.5	7	2.3	76	6.1	0.0034	30	0.1	<0.0001
**Mental health admission**		25	25.3	57	28.1	81	26.8	318	25.7		2345	6.0	
**Mental health admission and OST**		41	41.4	108	53.2	149	49.3	510	41.2		136	0.3	
**Mental health admission or OST**		70	70.7	168	82.8	237	78.5	904	73	0.0394	2511	6.4	<0.0001
**Cigarettes/day (vi)**													
None		23	23.2	29	14.3	52	17.2	287	23.2	0.0802	33699	85.6	<0.001
1–10		16	16.2	49	24.1	65	21.5	311	25.1		2974	7.6	
>10		46	46.5	112	55.2	158	52.3	605	48.9		2428	6.2	
Missing/not stated		14	14.1	13	6.4	27	8.9	35	2.8		266	0.7	
Total		99	100.0	203	100.0	302	100.0	1238	100.0		39367	100.0	
Any smoking during pregnancy		76	76.8	174	85.7	250	82.8	960	77.5	0.0364	5813	14.8	<0.0001
**Number of incarcerations (vii)**													
1		35	35.4	67	33.0	102	33.8	711	57.4	<0.0001	n/a	n/a	
2		14	14.1	39	19.2	53	17.5	256	20.7		n/a	n/a	
3		11	11.1	35	17.2	46	15.2	111	9.0		n/a	n/a	
4		12	12.1	15	7.4	27	8.9	67	5.4		n/a	n/a	
5		5	5.1	8	3.9	13	4.3	33	2.7		n/a	n/a	
6–10		17	17.2	32	15.8	49	16.2	55	4.4		n/a	n/a	
11–20		5	5.1	7	3.4	12	4.0	5	0.4		n/a	n/a	
**Total *N***		**99**	**100.0**	**203**	**100.0**	**302**	**100.0**	**1238**	**100.0**		**39367**	**100.0**	

(i) For each group, singleton births and first births during the study period (index births) only.

(ii) Births to women who were incarcerated for a minimum of five consecutive days during this pregnancy.

(iii) Births to women were not incarcerated during this pregnancy, but were incarcerated at another time during the study period.

(iv) A random 10% sample of births to women who were not incarcerated at all during the study period.

(v) One-way ANOVA and Bonferroni tests were used to test for differences between study and control groups in the distribution of maternal age; chi-square tests were used to look for differences between groups for all other maternal demographics.

(vi) Number of cigarettes smoked per day in the second half of pregnancy (from 20 weeks gestational age onwards).

(vii) Number of incarcerations of at least five days’ duration, 1998 to 2006.

**Table 2:** Pregnancy characteristics of women in the study (i), crude and adjusted odds ratio (OR) and 95% confidence intervals for odds ratios (95%CI) comparing prison pregnancies with controls

				**vs Prison controls**		**vs Community controls**	
		**Number**	**Percentage**	**Crude OR**	**Adjusted OR (95% CI)**	**Crude OR**	**Adjusted OR (95% CI)**
**1st antenatal visit >20 weeks gestation**							
Prison pregnancy (ii)		130	47.4	1.63	1.63 (1.24-2.14)*	6.82	4.05 (3.12-5.27)*
Prison controls (iii)		393	33.9	1.00	1.00		
Community (iv)		3928	10.1			1.00	1.00
**Not booked at admission**							
Prison pregnancy (ii)		55	19.0	1.31	1.17 (0.83-1.64)	8.92	3.24 (2.29-4.57)*
Prison controls (iii)		180	14.9	1.00	1.00		
Community (iv)		959	2.6			1.00	1.00
**Maternal diabetes mellitus**							
Prison pregnancy (ii)		1	0.3	0.45	0.43 (0.05-3.44)	0.55	0.41 (0.06-3.10)
Prison controls (iii)		9	0.7		1.00		
Community (iv)		237	0.6			1.00	1.00
**Gestational diabetes**							
Prison pregnancy (ii)		5	1.7	0.58	0.63 (0.24-1.62)	0.35	0.56 (0.23-1.39)
Prison controls (iii)		35	2.8	1.00	1.00		
Community (iv)		1799	4.6			1.00	1.00
**Maternal hypertension**							
Prison pregnancy (ii)		2	0.7	2.06	2.12 (0.39-11.65)	0.62	0.78 (0.19-3.31)
Prison controls (iii)		4	0.3	1.00	1.00		
Community (iv)		419	1.1			1.00	1.00
**Gestational hypertension**							
Prison pregnancy (ii)		8	2.8	0.77	0.80 (0.37-1.72)	0.41	0.49 (0.24-1.01)
Prison controls (iii)		42	3.5	1.00	1.00		
Community (iv)		2448	6.5			1.00	1.00

(i) For each group, singleton births and first births during the study period (index births) only.

(ii) Births to women who were incarcerated for a minimum of five consecutive days during this pregnancy.

(iii) Births to women were not incarcerated during this pregnancy, but were incarcerated at another time during the study period.

(iv) A random 10% sample of births to women who were not incarcerated at all during the study period.

(v) Logistic regression, adjusted for maternal age, parity, smoking, Aboriginal and Torres Strait Islander origin, mental health and/or drug and alcohol disorder.

*Result was significant (p0.05).

**Table 3:** Labour and delivery outcomes of women in the study

		**Pregnancy in prison (ii)**										
		**Birth in prison**		**Birth outside prison**			**Prison controls (iii)**			**Community controls (iv)**		
		***n***	***%***	***n***	***%***	**P value (v)**	***n***	***%***	**P value (v)**	***n***	***%***	**P value (v)**
**Onset of labour**												
	Spontaneous	64	64.6	156	77.6	0.5	866	71.0	0.5	24064	61.5	<0.0001
	Induced	22	22.2	28	13.9		232	19.0		10116	25.9	
	No labour	13	13.1	17	8.5		122	10.0		4924	12.6	
	Missing/not stated	0		0			0			8	0.0	
	**Total**	99	100.0	201	100.0		1220	100.0		39112	100.0	
**Presentation at birth**												
	Vertex	88	88.9	180	89.6	0.06	1118	91.6	0.9	35533	90.8	0.21
	Non-vertex	10	10.1	8	4.0		64	5.2		1581	4.0	
	Missing/not stated	1	1.0	13	6.5		38	3.1		1998	5.1	
	**Total**	99	100.0	201	100.0		1220	100.0		39112	100.0	
**Method of birth**												
	Normal vaginal	66	66.7	162	80.6	0.10	897	73.5	0.8	23669	60.5	<0.0001
	Instrumental vaginal	2	2.0	11	5.5		67	5.5		5126	13.1	
	Caesarean section	28	28.3	25	12.4		243	19.9		10191	26.1	
	Missing/not stated	3	3.0	3	1.5		13	1.1		126	0.3	
	**Total**	99	100.0	201	100.0		1220	100.0		39112	100.0	

(i) For each group, live born singleton births and first births during the study period (index births) only.

(ii) Births to women who were incarcerated for a minimum of five consecutive days during this pregnancy.

(iii) Births to women were not incarcerated during this pregnancy, but were incarcerated at another time during the study period.

(iv) A random 10% sample of births to women who were not incarcerated at all during the study period.

(v) Chi-square tests were used to test for differences between study and control groups in labour and delivery outcomes.

#### Maternal and neonatal outcomes

We used logistic regression to calculate the log odds of Caesarean birth, adjusting for known confounders. We used Cox regression analysis to calculate the relative risk of neonatal outcomes for each group, with both community controls and prison controls as the reference group and adjusting for known confounders (Table 4). For neonatal hospital admission and neonatal intensive care, we also adjusted for low birthweight and pre-term birth. We used univariate Cox regression analysis to test the significance of each covariate for each outcome, and included statistically significant covariates (*p* < 0.05) in the model for each outcome. There was a high correlation between study group membership and mental health disorders, drug and alcohol disorders and OST; therefore, we grouped all three into a single indicator. Cases with missing values were excluded from the regression analyses.

**Table 4:** Neonatal outcomes of babies in the study

			**Number**	**Crude rate**	**Crude RR**	**Adjusted RR (95% CI) (Prison controls = reference group)**	**Crude RR**	**Adjusted RR (95% CI) (Community = reference group)**
**Pre-term birth (gestational age <37 weeks)**								
Prison pregnancy (ii)			45	15.0	0.97	0.93 (0.67-1.28)	2.85	1.48 (1.07-2.03)*
Prison controls (iii)			188	15.4	1.00	1.00		
Community (iv)			2056	5.3			1.00	1.00
**Low birthweight (<2500 g)**								
Prison pregnancy (ii)			55	18.3	0.99	0.93 (0.69-1.24)	4.04	1.71 (1.28-2.30)*
Prison controls (iii)			225	18.4	1.00	1.00		
Community (iv)			1774	4.5			1.00	1.00
**Apgar score at 5 mins <7**								
Prison pregnancy (ii)			8	2.7	0.79	0.74 (0.35-1.57)	1.81	1.19 (0.57-2.49)
Prison controls (iii)			41	3.4	1.00	1.00		
Community (iv)			575	1.5			1.00	1.00
**Higher order resuscitation**								
Prison pregnancy (ii)			3	1.0	0.68	0.66 (0.20-2.24)	1.17	0.65 (0.20-2.10)
Prison controls (iii)			18	1.5	1.00	1.00		
Community (iv)			333	0.9			1.00	1.00
**All neonatal hospital admission (v)**								
Prison pregnancy (ii)			167	55.7	1.09	1.12 (0.94-1.33)	1.77	1.51 (1.29-1.78)*
Prison controls (iii)			622	51.0	1.00	1.00		
Community (iv)			12302	31.5			1.00	1.00
**5+ days in Neonatal Intensive Care Unit (v)**								
Prison pregnancy (ii)			51	0.2	0.96	0.93 (0.69-1.26)	3.64	1.45 (1.06-1.97)*
Prison controls (iii)			216	0.2	1.00	1.00		
Community (iv)			1826	0.0			1.00	1.00
**Optimum baby outcomes**								
Prison pregnancy (ii)			148	49.3	0.96	0.97 (0.81-1.16)	0.63	0.70 (0.60-0.83)*
Prison controls (iii)			630	51.6	1.00	1.00		
Community (iv)			30578	78.2			1.00	1.00
**Perinatal deaths (vi)**								
Prison pregnancy (ii)			2	0.7	0.46	0.43 (0.10-1.86)	1.06	0.56 (0.14-2.50)
Prison controls (iii)			18	1.5	1.00	1.00		
Community (iv)			246	0.6			1.00	1.00

(i) Except for perinatal deaths, live born singleton births and first births during the study period (index births) only. Analyses were adjusted for the confounding effects of maternal age, smoking, parity, Aboriginal and Torres Strait Islander origin, and any mental health admission/OST.

(ii) Births to women who were incarcerated for a minimum of five consecutive days during this pregnancy.

(iii) Births to women were not incarcerated during this pregnancy, but were incarcerated at another time during the study period.

(iv) A random 10% sample of births to women who were not incarcerated at all during the study period.

(v) Adjusted for pre-term birth, low birthweight, maternal age, smoking, parity, Aboriginal and Torres Strait Islander origin, and any mental health admission or OST.

(vi) Singleton births and first births during the study period (index births) only were analysed.

*Result was significant (p < 0.05).

#### ‘Optimum baby outcomes’

We created a composite indicator of ‘optimum baby outcomes’ for live-born, singleton babies in the study. Inclusion criteria:

• Gestational age ≥37 weeks;

• Birthweight ≥2500 g;

• Apgar score ≥7;

• No higher order resuscitation;

• No admission to Special Care Nursery or Neonatal Intensive Care Unit; and

• Survivor ≥ 28 days.

We used Cox regression analysis to investigate the likelihood of optimum baby outcomes for each group, following the same procedures as for other outcomes (Table 4).

## Results

### Maternal characteristics

Women in the prison pregnancy and prison control groups had similar socio-demographic characteristics, but were significantly different from women in the community control group on a broad range of socio-demographic and pregnancy risk factors (Table 1). Pregnant prisoners were, on average, 3.6 years younger than community controls (95% confidence interval (CI) 2.8-4.3 years), but only 0.7 years younger than prison controls (95% CI 2.44-3.20 years). Compared with prison controls, pregnant prisoners were no more likely to be parous, to be of higher parity, or to have been born in Australia. In contrast, pregnant prisoners were more likely to be Aboriginal and to have received inpatient treatment for mental health and/or drug and alcohol disorders, or OST, than women in the community (Table 1). Pregnant prisoners were more frequently incarcerated than prison controls. Of those in prison at the time of giving birth, 27.4% had five or more incarcerations during the study period, compared with 23.1% of pregnant prisoners who birthed in the community and 7.5% of prison controls (*p* <0.0001) (Table 1).

### Pregnancy characteristics

#### Antenatal care

The prison pregnancy group was much more likely than women in the community to have had limited or no antenatal care. Pregnant prisoners were over four times more likely than community controls to initiate antenatal care after 20 weeks of pregnancy (95% CI 3.1-5.3) and three times more likely not to be booked at the time of the birth admission (95% CI 2.3-4.6) (Table 2). Pregnant prisoners were also more likely than prison controls to initiate antenatal care after 20 weeks (adjusted odds ratio (OR) 1.63, 95% CI 1.24-2.14).

#### Maternal morbidity

Despite limited antenatal care, levels of maternal morbidity (gestational diabetes and pre-eclampsia) were lowest among pregnant prisoners. Pregnant prisoners were no more or less likely than community controls or prison controls to have pre-existing hypertension, diabetes mellitus, or gestational diabetes.

### Labour and delivery outcomes

Table 3 presents information about labour and delivery. The Caesarean section rate for birthing prisoners was comparable with community controls (28% and 26% respectively). Former pregnant prisoners were significantly less likely to have a Caesarean birth than birthing prisoners (adjusted OR 0.38 (0.21-0.70)), as were prison controls (adjusted OR 0.60 (0.38-0.96)). The highest rate of spontaneous onset of labour was found among former pregnant prisoners (77.6%). There was no significant difference between the groups in fetal presentation.

### Perinatal outcomes

#### Optimum baby outcomes

Pregnant prisoners were significantly less likely to have optimum baby outcomes when compared with community controls, (adjusted relative risk (RR) 0.70, 95% CI 0.60-0.83). However, there was no overall statistically significant difference between the prison pregnancy and prison control groups (adjusted RR 0.97, 95% CI 0.81-1.16). When prison controls were used as the reference group, older maternal age (0.94; 0.91-0.97), smoking (0.94; 0.91-0.97), and mental health or drug and alcohol disorder (0.89; 0.85-0.93) were predictive of poorer outcome. We also found no significant difference in the likelihood of optimum baby outcomes when birthing prisoners and former pregnant prisoners were compared with prison controls (adjusted RR birthing prisoners: 0.99 95% CI 0.75-1.3; former pregnant prisoners: 0.97 95% CI 0.78-1.2).

#### Neonatal hospital admission

Babies born to pregnant prisoners were significantly more likely to be admitted to hospital, and had a 50% greater risk of spending five or more days in intensive care, compared with babies born to community controls (Table 4). However, there was no significant difference between the prison pregnancy and prison control groups.

#### Other perinatal outcomes

For every outcome we investigated, babies born to women in the prison pregnancy group were significantly more likely to have poorer outcomes than community controls (Table 4). However, pregnant prisoners were not significantly more or less likely than prison controls to have poorer perinatal outcomes. Despite differences in their labour and delivery outcomes, there was also no significant difference in neonatal outcomes for birthing prisoners and former pregnant prisoners. The strongest predictors of neonatal outcome were mental health and/or drug and alcohol disorders, and smoking during pregnancy. There was no statistically significant difference between the groups in the risk of perinatal death, with maternal smoking the only significant factor in the model for this outcome (Table 4).

## Discussion

In this study, imprisonment during pregnancy did not improve maternal and perinatal outcomes for similarly disadvantaged women. Unlike previous studies [13], which found that imprisoned pregnant women had better perinatal outcomes than disadvantaged controls, our study found no evidence of any significant benefits for women or neonates. We attribute this to using controls that were themselves prisoners during the same time period, but not during pregnancy. Pregnant prisoners in our study were at the extreme end of the continuum of social disadvantage (Table 1) and were 12.6 times more likely to be of Aboriginal and Torres Strait Islander origin than women in the community. Furthermore, 78.5% had either a mental health and/or drug and alcohol disorder; we concluded that these morbidities, coupled with very high rates of smoking during pregnancy, primarily accounted directly or indirectly for their significantly higher risk of poorer maternal and perinatal outcomes. The prison control group shared these characteristics, and our study demonstrates that interventions received whilst pregnant in prison were not successful in mitigating poorer outcomes.

Low birthweight was investigated in the only other study to use incarcerated women as a control group [11]. This US study, which also compared incarcerated pregnant women (n = 168) with women imprisoned at a time other than during their pregnancy, found that pregnant prisoners were less likely than prison controls to have low birthweight babies (adjusted OR 0.54, 95% CI 0.32-0.93). The same study concluded that following adjustment for social disadvantage, pregnant prisoners were not significantly more or less likely to have low birthweight babies than women in the community. Our study adjusted primarily for co-morbidities and found that there was no significant difference between the prison pregnancy and prison control groups (adjusted RR 0.93, 0.69-1.24), but that pregnant prisoners were significantly more likely than community controls to have a low birthweight baby (adjusted RR: 1.71, 95% CI 1.28-2.30). The US study also found that birthweight increased with number of weeks of pregnancy in prison [11]. The ‘high-volume, short-term’ nature of women’s imprisonment in Australia [21] may have diluted our findings. Shorter lengths of stay of women in Australian prisons may provide insufficient opportunity to improve birthweight. Mental health, including substance use disorders, was highly significant in all of our regression models, but was not included in the aforementioned study [11]. It is well established that poor maternal mental health is a risk factor for poorer neonatal and infant outcomes, and this risk increases during pregnancy and the postnatal period [22]. It is evident that consideration of maternal mental health is essential to any study of the maternity outcomes of prisoners, and indeed to any intervention with this group.

The literature is equivocal on incarceration and pre-term birth, with a small US cohort study of 69 prisoners who received prenatal care from a single medical centre finding no difference from community controls. In contrast, two studies found pregnant prisoners were more likely to have pre-term births than community controls [8,9]. Whilst this finding held true in our study, there was no statistically significant difference between the prison pregnancies and prison controls. Apart from these two studies, no others have used disadvantaged controls to investigate pre-term birth.

We found a higher rate of neonatal hospital admission and length of stay in SCN/NICUs for babies born to pregnant prisoners, when compared to community controls. This persisted after controlling for known risk factors. This finding conflicts with an earlier US study that found no difference between prisoners and hospital controls, and reflects the potential burden of mental health and/or drug and alcohol disorders found among prisoners [9]. We found no significant difference when comparing prison pregnancies with prison controls.

Our study found that birthing prisoners had rates of Caesarean section and induction of labour that were comparable with the community, whilst former pregnant prisoners and prison controls tended to be more likely to have a spontaneous vaginal birth. The post-release period is one of high risk, when contact with health services is likely to be erratic [2]. It is possible that women in the latter two groups presented at a more advanced stage of labour. Imprisoned women and their clinicians, given the challenges of birthing spontaneously in a prison setting, may favour increased intervention and ‘control’ over birth. Data on place of birth would assist in interpreting these findings.

Our study found no evidence of a statistically significant difference in the likelihood of fetal death between the prison or control groups. However, the number of deaths was small; and of the two fetal deaths in the prison pregnancy group, neither was to a birthing prisoner. A UK study suggested that there might be a reduced fetal mortality rate among imprisoned women, compared with births in families where at least one parent was under the Probation Service; however, a more carefully controlled study was recommended [10]. This study provided the only evidence for the subsequent systematic review that suggested that imprisoned women might be less likely to have a stillbirth than similarly disadvantaged controls [13]. This was not our finding and no other studies have examined this outcome.

Internationally, there remains wide variation in prison maternity care. Despite the availability and proximity of prison medical facilities, women in our study who were pregnant in prison were more than twice as likely to initiate antenatal care after 20 weeks’ gestation compared with prison controls. One US study reported that prenatal education, childbirth instruction, and general health education classes were mandatory for pregnant prisoners [9]. A 2006 report by the UK’s Maternity Alliance found ‘a high level of variation in the quality of antenatal care between prisons… The good practice found in some establishments was not replicated in others’ [23], and a report by the UK National Childbirth Trust (NCT) noted that prison routines and poor communication interfered significantly with pregnant women’s participation in antenatal classes, during a pilot study at one of England’s largest women’s prisons [24].

This study has a number of strengths. It is population based and uses women with a history of incarceration as a control group to investigate a range of important maternal and perinatal outcomes. Additionally, the study design incorporated linkage of data on mental health and drug and alcohol disorders allowing us to control for these highly significant confounding factors. However, for perinatal deaths and maternal morbidity, the small population of incarcerated pregnant women likely contributed to the lack of statistically significant findings. Information about socio-demographic factors that have a well-established correlation with both maternity outcomes and female imprisonment, including maternal education, employment, and marital status as well as health risk factors such as sexually transmitted infections, blood-borne viruses, previous pregnancies and maternal weight, would assist with the interpretation of findings. More robust data about incarceration histories, police and Juvenile Justice involvement would enhance our understanding of the interaction between health and social factors in maternity outcome for these women, and help to signal opportunities for intervention. Data about legal status (remand or sentenced) would indicate the type of accommodation and level of access to services women may have had whilst imprisoned. Data on the timing of onset of antenatal care would assist in understanding the nature of the care women had received. Further detailed analyses will be undertaken to determine whether the number of days’ incarceration during pregnancy, and gestation at incarceration, are related to differences in outcome for women and babies.

## Conclusions

The notion that prisons are “therapunitive” environments is not supported by empirical evidence [25]. Prison health services are ill equipped to mediate the cumulative disadvantages, both health and social, to which their clients are exposed throughout the life-course. Post-release, most return to ‘liminal, marginal spaces’, which provide the context for re-incarceration [2].

## Abbreviations

NSW: New South Wales; US: United States; UK: United Kingdom; MDC: Midwives Data Collection; OIMS: Offender Integrated Management System; APDC: Admitted Patient Data Collection; PHDAS: Pharmaceutical Drugs of Addictions System; OST: Opioid Substitution Therapy; SCN: Special Care Nursery; NICU: Neonatal Intensive Care Unit.

## Competing interests

The authors declare that they have no competing interests.

## Authors’ contributions

MHL and EAS conceived and supervised the study, contributed to the interpretation of data and reviewed the manuscript. LH obtained and prepared the linked data set for analysis, contributed to the interpretation of data and reviewed the manuscript. JRW conducted the analysis and interpretation of data, and wrote the manuscript. All authors read and approved the final manuscript.

## Pre-publication history

The pre-publication history for this paper can be accessed here:

http://www.biomedcentral.com/1471-2393/14/214/prepub

## Supplementary Material

Additional file 1 Overview of data sets included in the linkage. Click here for file
