# Supplementary material for: Pregnancy, prison and perinatal outcomes in New South Wales, Australia: a retrospective cohort study using linked health data
Source: BMC Pregnancy Childbirth. 2014 Jun 27;14:214. doi: 10.1186/1471-2393-14-214 (PMC4099381; doi:10.1186/1471-2393-14-214)
Supplement: Additional file 1 — Overview of data sets included in the linkage. [file 1471-2393-14-214-S1.docx]

# Additional File 1

##### Data sources

The Midwives Data Collection (MDC) is a population-based surveillance system including all births of at least 400g birthweight or at least 20 weeks gestation in New South Wales (NSW) public and private hospitals, as well as home births. Information is recorded by midwives or medical practitioners, and includes demographic, medical and obstetric information on the mother and information on the labour, delivery and condition of the infant. The Offender Integrated Management System (OIMS) is a system used by Corrective Services NSW to support case management of prisoners. It contains records relating to location and transfer history, classification, security, self-harm, demographics, and biometric identification for all persons of 18 years or older that have spent time in prison in NSW. The Admitted Patient Data Collection (APDC) is an administrative census of services for patients admitted to public hospitals, public psychiatric hospitals, public multi-purpose services, private hospitals and private day procedure centres in NSW. It contains information on patient demographics, diagnoses, procedures, and external causes, individually coded using ICD10-AM codes, for each hospital admission. Only records with specified ICD10-AM diagnoses indicating substance use or psychiatric illness, or flagged as having been admitted to a psychiatric ward, were within the scope of the linkage. The Pharmaceutical Drugs of Addiction System (PHDAS) is register of authorities to dispense drugs of addiction.

##### Record linkage

The NSW Centre for Health Record Linkage (CHeReL) conducted a probabalistic linkage of named data. The CHeReL undertakes annual linkage of NSW health data to create a ‘master linkage key’ that indicates where records for the same person can be found in the different health databases. Records of for 10,372 women aged 18-44 years who spent time in prison between 1 January 1998 and 31 December 2006, were extracted from the OIMS and 3085 were linked with the record of a birth between 1^st^ July 2000 and 31^st^ December 2006.

External person identifiers supplied by OIMS and PHDAS were matched with the CHeReL master linkage key data in two stages. First, OIMS and PHDAS identifiers were linked with maternal MDC and women’s APDC record identifiers. The linked records, and records for a randomly selected ten per cent sample, were returned to the MDC data custodian, who provided identifiers for the babies born to these mother for a second linkage. Identifying data from APDC records for babies admitted as neonates were matched with the MDC baby identifiers.

A unique study person number was added to the linked records returned to each data custodian. All other identifying information was removed. Study data were added and de-identified datasets were provided to the researchers. This was re-assembled to form a single dataset in which all records for each mother were linked together.

Results of the linkage process can be found in a separate paper.
